# Emergency Medicine Residents on Electronic Medical Records: Perspectives and Advice

**DOI:** 10.7759/cureus.4027

**Published:** 2019-02-07

**Authors:** Maxwell A Hockstein, Sara N Pope, Kayla Donnawell, Summer A Chavez, Lipika Bhat

**Affiliations:** 1 Emergency Medicine, Emory University, Atlanta, USA; 2 Emergency Medicine, UH Regional Hospitals, Richmond Heights, USA; 3 Emergency Medicine, St. Louis University, St. Louis, USA; 4 Emergency Medicine, Medstar Georgetown University Hospital, Washington, USA; 5 Emergency Medicine, University of Kentucky, Lexington, USA

**Keywords:** electronic medical records, emergency medicine residency

## Abstract

Given the near-universal implementation of electronic medical records (EMRs) in emergency departments (EDs), emergency medicine (EM) residents spend significant time interfacing with EMRs without any established national curriculum to learn best practices. While EMRs have the potential to increase physician efficiency and improve the quality of documentation, they have also been cited as a factor in physician burnout. Understanding the target audience of the EMR, knowing what and when to chart, and practicing time-saving strategies can streamline the process of charting. We review the literature on the current state of EMR documentation by residents and provide recommendations for best practices.

## Introduction and background

Electronic medical records (EMRs) have been in existence for decades [1]; however, their prevalence in healthcare facilities has increased dramatically only over the past few years. One driving influence was the Health Information Technology for Economic and Clinical Health (HITECH) Act in 2009 [2]. Centers for Medicare and Medicaid Services (CMS) promoted HITECH, specifically the “meaningful use” of EMRs, by financially rewarding institutions that proved that EMRs were being used to improve patient care. Most academic medical centers use the more popular EMRs, namely, Epic (Epic Systems, Verona, WI), Cerner (Cerner Corp., Kansas City, MO), and Allscripts (Allscripts Healthcare Solutions, Chicago, IL) [3].

As of 2017, 96% of non-federal hospitals possessed a certified EMR system, a percentage that has been relatively constant since 2015 [4]. Most emergency medicine (EM) residencies utilize EMRs. In fact, the Fellowship and Residency Electronic Interactive Database (FREIDA) no longer polls EM residencies if they use EMRs because so many have responded “yes” that it was no longer a useful metric [5]. Regardless of the software, a significant amount of time is spent on the EMR compared to the amount of time spent at the bedside. One study conducted at a community emergency department (ED) cited that an average of 44% of a practitioner’s shift was dedicated to data entry and only 28% dedicated to patient contact [6]. Similar percentages have been reported across medical specialties [7], resulting in physician frustration. Moreover, such frustration with the clerical burden places physicians at an increased risk for burnout [8], an already pervasive problem in healthcare professions, especially EM [9].

## Review

Interested stakeholders

The information within the medical record is important to multiple groups: clinicians to provide effective clinical care, medical coders for billing, researchers for scientific studies, advocates for quality improvement, and patients interested in their healthcare information. First, healthcare provider charting is important so that other healthcare providers will have an accurate and comprehensive image of the patient’s visit and implications for his/her overall health. Second, many emergency physician groups link provider reimbursement to relative value units (RVUs), which is derived from ED visit EMR documentation. As such, it should be clear to medical coders as to what services and procedures were implemented as well as what conversations occurred. And third, one of the realities of medical charting is that it is subject to review during both quality improvement initiatives and legal proceedings. Therefore, decisions made during a patient’s ED stay should be explained in the EMR text. Justifications should include all medical decision making that helped guide management. Though a few EMRs have integrated clinical decision rules (e.g., the pulmonary embolism rule-out criteria (PERC)), it is helpful to include a concise medical decision-making summary of the relevant ones. Documenting what was offered and then decided, especially if shared decision making was used, is integral in any chart review. Finally, patients are taking a greater role in accessing their protected healthcare information.

Importance for residents

EM residents spend a great deal of time with the EMR – performing chart review, data and order entry, note writing, disposition planning, and handoff. The known benefits of EMRs, such as accessibility outside the clinical area, computerized order entry, referrals, and centralization of patient information, contrast the risks of less direct patient interaction, burnout, and inaccurate charting. Interestingly, however, some studies suggest that patients do not perceive the physician’s focus on the EMR to impact the patient-doctor relationship [10]. There is little data to support that EMRs change resident productivity [11]. In addition, the advent of pre-populated notes and phrases, designed to improve efficiency and optimize levels of service, may inadvertently document exams not performed. Despite the intent to make charting more efficient, one study showed that EMR documentation actually took longer to complete than paper charting [9].

Knowing what and when to chart, EMR shortcuts, and documentation tips are frequent topics of conversation throughout residency; yet, there is no formalized national curriculum on EMR use for EM residents, to our knowledge. Moreover, there are even fewer opportunities for students to participate in chart documentation [12]. Only recently were medical students given permission by CMS to document in the chart without the attending physician duplicating their charting [13]. In many residency programs, education about chart documentation involves trial, error, feedback, and repetition of the same. Instead, charting should be approached methodically, similar to a procedural skill. Step-by-step instruction using video modules, for instance, has been shown in other training specialties to improve efficiency and completeness [14]. The degree of EMR complexity currently requires dedicated training to navigate the intricacies of the system.

Ensuring optimal billing of my chart

The adage, “If it wasn’t documented, it wasn’t performed,” has been revised to “If it wasn’t documented, it cannot be billed.” When coders review charts, they use specific criteria, such as those from CMS, to determine which billing codes are most appropriate. Documentation that supports the treatment and care of a medically complex patient receives a higher level of service and ultimately greater reimbursement than an uncomplicated patient with a straightforward condition. The three main components that dictate the Evaluation and Management (E/M) level of service are the history, physical examination, and medical decision making. One commonly accepted method of scoring the amount of history and exam in the medical record is the Medicare 1995 Documentation Guidelines. Although these guidelines are likely to change in the near future [15], both the 1995 and 1997 documentation guidelines are both in use depending on departmental preference. Though there is no formal billing curriculum, one potential model to teach billing and coding is to have residents participate in an exclusively billing and coding shift which also would likely increase the efficiency of coders [16]. A complete discussion of billing is beyond the scope of this paper. There are several references available on how to optimize documentation for billing purposes [17].

Strategies and tips on more efficient charting

Avoid Redundancy

The EMR serves as a hub for interactions separate from physician documentation, at times eliminating the need for additional documentation from the physician. In all EMRs, physician orders are required to be time-stamped and recorded in the patient’s record. Restating these in the physician note may not add any clarity; however, a brief mention of the rationale behind placing the orders provides information that is helpful in better understanding the patient’s encounter.

Similarly, some EMRs import problem lists, allergies, vital signs, and test results, thus allowing the physician to comment on the significance of the results without having to restate the raw data. In fact, there is data to suggest that even when included in a note, many physicians skip over imported data and focus on the free-texted impression and plan [18]. We believe that often a lack of understanding of what is necessary to include in the chart turns into repetitive statements with less focus on providing insights into a resident's decision-making process. Residents may save time by focusing on answering questions of “why” rather than redundant charting. Some EMRs allow for the importation of photos of physical exam findings, this may help limit descriptive text.

Text-expander Shortcuts

Decreasing the number of keystrokes saves time. “Dot phrases” or “macros” are useful text-expander tools for repeatedly documented information. For instance, a text-expander can be used to write return precautions for a patient with undifferentiated abdominal pain who did not undergo computed tomography (CT) imaging or for a patient with an uncomplicated asthma exacerbation. These shortcuts can incorporate clinical decision tools, which are frequently used within the medical decision-making section, such as the PERC rule. For procedure documentation, using a text block with specific language including relevant elements not only saves time but also facilitates accurate billing, because the phrases align with what the coders are looking for.

Text blocks can be customized to the individual needs of patients. An example might include specifying the number of days before suture removal. Another example includes documenting the management and discussion of a patient who leaves against medical advice. Standard departmental policies and language can be saved as a text block and then edited to discuss the clinician-patient discussion in more detail. This approach helps protect the clinician and department in such higher-risk patient encounters.

With the flexibility to customize often-repeated phrases, there may be some confusion as to the contents and formatting, especially for trainees early-on in their career. Standardizing templates for notes allows the physician trainee to grasp the fundamental components of documenting the patient encounter. While these electronic phrases have been passed from resident to resident, there may be a role in cataloging these text-expanders and integrating them into workflows, elevating the EMR to the next level. This is an area that EMR vendors can provide assistance with. Text-expander shortcuts should be scrutinized for generalizability. Despite their evident utility, text-expander shortcuts have the danger of inadvertently documenting conditions not present and exams not performed.

Documentation Aids 

Many clinical sites use aids such as scribes and voice recognition software to improve charting efficiency. Because such documentation aids come with variable financial and administrative costs, departments and hospital groups should evaluate and weigh these factors before implementing them.

Use of scribes for documentation occurs in more than 1,000 hospitals across 44 states and estimates project that by 2020, there will be one scribe to every nine physicians, or an estimated 100,000 scribes [19]. In an outpatient setting, a randomized control trial demonstrated that scribes improved physician satisfaction, increased face time with patients, increased charting accuracy, decreased total charting time, and increased chart completion compliance within 48 hours. Patient satisfaction was interestingly not affected in this study [20]. In an academic setting, scribes were found to decrease documentation time by 36%, increase direct patient contact time by 30%, and increase hourly RVUs by 5.5% [21]. A potential area of future study is to examine how ED scribes impact resident or medical student teaching.

Voice recognition software has become increasingly portable and popular in EDs. In a direct comparison of voice recognition software and transcription services, one group found voice recognition to be superior given the shorter turnaround time and decreased transcription costs [22]. Another study found a decreased number of physician interruptions when voice recognition software was used in place of traditional typing-based charting [23].

Despite these potential benefits, voice recognition software, regardless of brand, occasionally transcribe nonsensical sentences. Most of the literature surrounding voice recognition software is from data that examine the transcription of radiologic studies. In one such study, 17.72% of the dictations had at least one error and 4.23% of the dictations had significant or very significant errors [24]. Another study even found an increase in dictation editing time compared to not using voice recognition [25]. All documentation, no matter how they are transcribed, should be proofread.

Timely Documentation

Not only should documentation be accurate, but it also should be completed in a timely fashion. A deliberate practice plan should thus be implemented early during residency training on timely documentation. Efficient workflow habits allow senior residents to balance documentation requirements with the additional responsibilities of caring for more critically ill patients and supervising trainees in the department.

One possible on-shift routine may be to see one to two patients, place orders, and while still at the computer, start writing the history of present illness (HPI), review of systems (ROS), physical exam, and differential diagnosis before signing up for a new patient. Later, placing discharge or admission orders should trigger a reminder to complete the medical decision-making section for that patient at the completion of the patient encounter.

Although the unpredictability and constant distractions in the ED often prevent a structured charting routine, we suggest avoiding waiting until the end of the shift to begin charting. While there is often pressure to promptly see newly arrived patients, a good habit is to update charts after every few patients while they are still fresh on one’s mind. Five different patients with abdominal pain are harder to differentiate at the end of a shift than immediately following each encounter.

A time-saving strategy is to write the note while in that patient’s room. While this could hinder communication, in an increasingly electronic-dependent society, it may not have a significant impact on patient satisfaction [10,26]. Real-time documentation also has the benefit of potentially increasing completeness on complex patients, such as trauma resuscitations [27]. If available, mobile charting stations allow the physician to chart in the EMR without having to walk back to a central workstation after each encounter. Additionally, structured data-entry forms may facilitate point-of-care documentation and increase the number of charts that are completed on time [28]. It should be noted that CMS does not explicitly give a deadline for charting after a patient encounter. It ambiguously recommends that the chart should be completed “as soon as practicable” [29].

Need for a curriculum

Despite the widespread use and amount of shift-time dedicated to EMRs, there is no formalized national curriculum dedicated to their best use. Preliminary data shows that personalized feedback improved resident documentation [30]. An EMR curriculum should focus on the creation, maintenance, and shareability of templates, optimized for efficient, accurate, and billable documentation. As chart requirements vary from department-to-department, this could make the establishment of such a curriculum challenging. The identification of a local documentation champion or medical informaticist helps facilitate local best practice. Once a physician has a template that serves its intended purposes, it is advisable to keep a plain-text version of it in their personal files as it is difficult to export templates from EMRs. 

## Conclusions

Novel technologies are created every day and, as emergency physicians, we are asked to adapt to these new advances. With laws like HITECH and the American Recovery and Reinvestment Act, EMRs are here to stay and will continue to be an integral part of our daily practice. Becoming efficient in charting is as important as learning any bedside procedure considering the amount of time spent with the EMR. By understanding the purpose of, and value in, charting we can better understand the pertinent information that should be included in the chart. By avoiding charting redundancies, using text-expander shortcuts, implementing documentation aids, and optimizing our charting workflow for timely documentation, we can become more efficient physicians who will be able to practice medicine, while spending more time with patients. The creation of an EMR curriculum will likely improve our understanding of documentation hurdles and improve the documentation quality of our specialty.
